# Diagnosis of Weissellosis in Cultured Rainbow Trout in Costa Rica Further Evidence Emergence of *Weissella tructae* as Important Pathogen of Salmonids in Latin America

**DOI:** 10.1155/tbed/4690289

**Published:** 2026-06-15

**Authors:** Esteban Soto, Carolina Elizondo, Zeinab Yazdi, Alvin Camus, Kim Li Jacobsen, Jefferson Yunis-Aguinaga, Paola Barato, Mónica Damme, Dennis Fuentes, Fernando Aguilar, Eunice Víquez-Ruiz, Francine Pereira, Marco Medina-Morillo, Gustavo Rojas, Elías Barquero-Calvo, Luke R. Iwanowicz, Timothy J. Welch

**Affiliations:** ^1^ Department of Medicine and Epidemiology, School of Veterinary Medicine, University of California-Davis, California, 95616, USA, ucdavis.edu; ^2^ SENASA, Ministerio de Agricultura y Ganadería, Heredia, Costa Rica, mag.go.cr; ^3^ Department of Pathology, College of Veterinary Medicine, University of Georgia, Athens, Georgia, 30602, USA, uga.edu; ^4^ Universidad Científica del Sur, Lima, Peru, cientifica.edu.pe; ^5^ Instituto del Mar del Perú (IMARPE), Callao, Peru; ^6^ Corporación Patología Veterinaria, CORPAVET, Bogotá D.C., Colombia, unal.edu.co; ^7^ Truchas Reales de Costa Rica S.A., San Jose, Costa Rica; ^8^ INCOPESCA, Instituto Costarricense de Pesca y Acuicultura, Puntarenas, Costa Rica; ^9^ Programa de Investigación en Enfermedades Tropicales, Escuela de Medicina Veterinaria, Universidad Nacional, Heredia, Costa Rica, una.ac.cr; ^10^ National Center for Cool and Cold Water Aquaculture, USDA-ARS, Kearneysville, West Virginia, USA, usda.gov

**Keywords:** emergent, salmonids, sepsis, *Weissella*

## Abstract

Weissellosis, caused by the Gram‐positive (GP) lactic acid bacterium *Weissella tructae*, is an emerging disease of farmed rainbow trout (*Oncorhynchus mykiss*) reported in Asia, South and North America. In this study, *W. tructae* was isolated from diseased cultured trout in Costa Rica on four facilities with mortalities seen predominantly in sub‐adult (100–700 g) and adult fish (>2 kg) during April and September 2024 (rainy season). Losses occurred at seasonally high temperatures (>18°C) with clinical signs and gross changes including high morbidity and mortality, lethargy, abnormal swimming, exophthalmia, and hemorrhagic lesions in external and internal tissues consistent with bacterial sepsis. Whole‐genome sequencing and phylogenomic analysis of isolates from Costa Rica (*n* = 4), Peru (*n* = 3), and Colombia (*n* = 2) revealed >99.99% genetic identity to each other and isolates from Brazil, Mexico, Colombia, and the United States. Western blot analysis using antibodies against *W. tructae* isolates from the Eastern United States, showed the isolates are antigenically similar to those from Costa Rica. These results provide evidence that the same pathogenic strain is causing outbreaks from the United States to Peru, suggesting a monovalent vaccine could protect trout farms across the Americas. To better define the role of temperature in weissellosis outbreaks, laboratory‐controlled challenges were performed at 13 and 18°C using intracelomic (ICe) injection and cohabitation models. Rainbow trout ICe shedders were cohabitated with naïve fish (cohabitants) for 21 days. Injected trout maintained at 13°C experienced mortalities of 40%; however, ~89% (16/18 fish) remained infected at the end of the challenge. No mortality occurred in cohabitants at 13°C, and only 1/30 survivors (~3%) showed evidence of infection. In contrast, mortality in injected shedders and cohabitants exceeded 96% (29/30 injected fish) and 16% (5/30 cohabitants) at 18°C, respectively. Persistent infection was observed in 100% (1/1 injected) and 4% (1/25 cohabitant) of fish surviving challenge at 18°C, confirming a significant role for temperature in the pathogenesis of weissellosis.

## 1. Introduction

Aquaculture is one of the world’s fastest‐developing food production sectors. In Latin America, aquaculture is dominated by the production of Atlantic salmon (*Salmo salar*) in Chile [1], as well as tilapia (*Oreochromis* spp.) and shrimp (*Penaeus* spp.) in Brazil and Ecuador [2, 3]. However, production of trout in freshwater has also increased in Latin American countries in North, Central, and South America [4]. In Peru, the second highest producer of rainbow trout (*Oncorhynchus mykiss*) after Chile, production reached 61,573 tonnes in 2022, making this species the most important cultured fish in the country [5, 6]. The majority of this production is intended for local consumption [7].

Infectious disease is a primary factor constraining sustainable expansion of aquaculture, where intensive culture practices have facilitated the emergence and rapid dissemination of several potentially devastating pathogens. Amongst the emerging diseases in Latin American rainbow trout aquaculture, lactococcosis and weissellosis have gained attention due to their association with high mortality and recent expansion to several countries on the continent [8, 9].

The first report of weissellosis in 2009 described an outbreak of bacterial septicemia at a commercial rainbow trout facility in China [10]. The disease is now recognized as an emerging issue in Asia, with outbreaks recently reported in Japan [11] and Vietnam [12]. In the Americas, the first reported outbreaks occurred in Brazil in 2008 and 2009 [8]. Not long after this, the disease was reported in North Carolina, USA [13], Central Western Mexico (2015) [14], and central Colombia [15].

Peru, one of the main Latin American producers of rainbow trout, has documented *Weissella tructae* outbreaks affecting aquaculture facilities in Lake Titicaca and Lake Arapa since 2018. The primary production system in this region utilizes floating cages (150–200 m^3^) with stocking densities of 5–10 kg/m^3^. Affected farms incur *Weissella*‐associated mortalities of up to 60%, with the highest incidence during the summer months (December–April) [16].

In Colombia, rainbow trout is the third most important freshwater aquaculture species, accounting for ~15% of national production, following tilapia (*Oreochromis* spp.; 52%) and cachama (*Piaractus brachypomus*; 17%). Since the first outbreaks of weissellosis in 2016, this disease has become one of the most severe challenges facing Colombian trout farming. Reported cumulative mortalities range from 32% to 70% and are largely associated with elevated water temperatures (>18°C). Current control strategies include the use of an autogenous vaccine implemented since 2024, oral supplementation with benzoic acid (75%), *Curcuma longa* (10%), and *Cinnamomum verum* (2.5%), as well as enhanced biosecurity measures and reduced stocking densities [15, 17].

In Costa Rica, Nile tilapia (*Oreochromis niloticus*) is the most widely cultured fish species in addition to spotted rose snapper (*Lutjanus guttatus*) and rainbow trout. While much of the country’s trout production is for recreational purposes, rainbow trout aquaculture provides an important protein source for rural communities. In this study, *W. tructae* infections were diagnosed for the first time in cultured trout in Costa Rica. We report findings of comparative genome and western‐blotting immunoassay analyses of *W. tructae* recovered from trout cultured in Colombia, Peru, Brazil, and the United States, indicating a high degree of similarity among isolates from the different countries. Additionally, laboratory‐controlled challenges by intracelomic (ICe) injection and cohabitation confirm the importance of temperature in the pathogenesis of weissellosis in rainbow trout.

## 2. Materials and Methods

### 2.1. Case Presentations and Fish Sampling

Acute to subacute mortality was reported in 2023 and 2024 in four distinct rainbow trout aquaculture facilities in Costa Rica (Figure 1 and Table 1). Complete postmortem examinations were performed on 5–10 fresh‐dead rainbow trout from each facility within 24 h of death by fish veterinarians.

**Figure 1:**
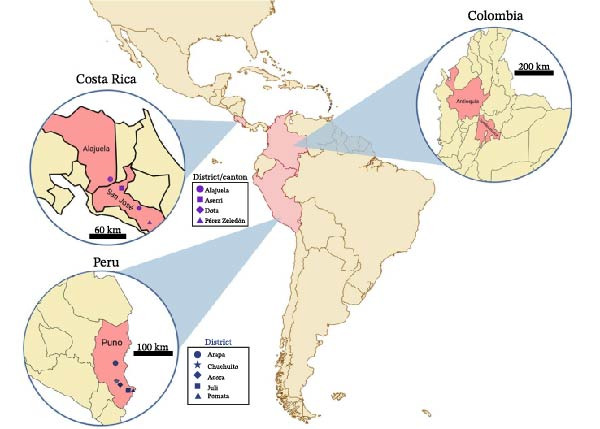
Map of Latin America indicating the location of weissellosis outbreaks in rainbow trout in Costa Rica (*n* = 4), Peru (*n* = 3), and Colombia (*n* = 2) from which samples were evaluated.

**Table 1:** Metadata of *Weissella tructae* isolates recovered in different aquaculture facilities during outbreaks of disease.

Isolate ID/date of isolation	Region of facility (country, province/city)	Water source	Aquaculture modality	Water temperature during outbreak	Stocking density during outbreak	Fish weight during outbreak	Percent mortality at end of outbreak
Costa Rica							
R24462/08‐2024	San Gerardo de Dota	Surface	Raceways	14–15°C	40–45 fish /m^3^, (~22,000 animals per raceway)	350 g	80%
R24464/08‐2024	Cedral, Perez Zeledón	Surface	Raceways	19.4–21.9°C	40–45 fish /m^3^, (~22,000 animals per raceway)	350 g	80%
R24466/11‐2024	Alajuela	Surface	Tanks	15–19°C	30 peces /m^3^	600–700 g	50%
R244610/08‐2024	Aserri	Surface	Lake	17–20°C	15 peces /m^3^	100 g	40%
Colombia							
R24493/10‐2022	Antoquia	Surface	Raceways	17–18.5°C	Not available	100–400 g	32%
R24492/08‐2019	Cundinamarca	Surface	Raceways	17–22°C	Not available	100–400 g	50%
Peru							
R250725/04‐2022	Lake Titicaca (Juli, Puno)	Surface	Lake floating cages	>15°C	5–10 kg/m^3^	>80 g	~ 60%
R25071/02‐2020	Lake Titicaca (Juli, Puno)	Surface	Lake floating cages	>15°C	5–10 kg/m^3^	>80 g	~ 60%
R25075/11‐2018	Lake Titicaca (Faro/Pomata)	Surface	Lake floating cages	>15°C	5–10 kg/m^3^	5‐fry pool	Subclinical (0%)

### 2.2. Necropsy and Histopathology

Representative samples of liver, kidney, heart, spleen, eye, gill, and intestine were collected from diseased fish and fixed in 10% neutral buffered formalin (NBF) for at least 48 h. For histopathologic evaluation, fixed tissues were processed routinely, embedded in paraffin, sectioned at 5 µm, and stained with hematoxylin and eosin stain (H&E) [18]. Selected tissues were stained with a modified Brown and Hopps tissue Gram stain.

### 2.3. Bacterial Isolation and Culture

For bacterial isolation, swabs of posterior kidney, eyes, and brain were aseptically collected and streaked onto Columbia agar supplemented with 5% sheep blood (Neogen, USA) and incubated at 25 or 30°C for up to 5 days. Pure or dominant bacterial colonies were harvested and preserved in a solution containing 10% skimmed milk (Sigma–Aldrich, USA) and 20% glycerol (Sigma–Aldrich, USA) and stored at −80°C until further use.

### 2.4. Morphological, Physiochemical, and Antimicrobial Susceptibility Analysis

Thawed bacterial isolates were revived on Columbia agar incubated at 25°C for 24–48 h. Colony morphology was documented, and Gram staining was performed following standard protocols. Using fresh colonies, catalase and oxidase activity were evaluated by applying 3% hydrogen peroxide and using oxidase test strips (Oxoid, United Kingdom), respectively. A previously identified *Aeromonas cavieae* by whole‐genome sequencing and analysis (data not shown) was used for comparison purposes. Biochemical characterization utilized the VITEK 2 Compact system (bioMérieux, France) with Gram‐positive (GP) identification cards, following the manufacturer’s instructions. Identification was further confirmed by matrix‐assisted laser desorption/ionization time‐of‐flight mass spectrometry (MALDI‐TOF MS) using the Bruker MALDI Biotyper Sirius system (San Jose, Costa Rica), following the manufacturer’s guidelines for bacterial identification. Although the Bruker Biotyper database includes several species of the genus *Weissella*, *W. tructae* is not currently represented in the commercial database. Consequently, species‐level identification is not supported for this organism. According to Bruker criteria, scores ≥2.0 indicate confident genus‐ and species‐level identification, whereas scores between 1.7 and <2.0 permit reliable identification only at the genus level. In this study, the isolates yielded scores between 1.7 and <2.0 for the genus *Weissella*; therefore, the MALDI‐TOF MS results were considered reliable at the genus level only [19].

Antimicrobial susceptibility was compared between four selected *W. tructae* isolates recovered from different outbreaks and farms in Costa Rica using the Sensititre Avian AVIAN1F AST Plate system (Thermo Fisher Scientific, USA). There are no established and validated methods for antimicrobial susceptibility testing for *W. tructae*. As such, manufacturer’s protocol and CLSI recommendations for *Lactococcus* were followed with modifications [20–22]. Briefly, a 0.5 McFarland suspension of each isolate was diluted 1:1000 (∼1.5 × 10^5^ colony forming units [CFU]/mL) into a cation‐adjusted Mueller‐Hinton broth (CAMHB) with 5% lysed horse blood. A total of 50 μL bacterial suspension was distributed into Sensititre plate wells, incubated at 28°C for 48 h, and the minimal inhibitory concentration for each antimicrobial was determined by the absence of bacterial growth in the relevant wells. *Escherichia coli* ATCC 25922 was used as a quality control strain and tested under the same conditions as the *W. tructae*.

### 2.5. Molecular Identification and Sequencing

Genomic DNA was extracted from pure bacterial isolates recovered from outbreaks in Costa Rica (*n* = 4) and Peru (*n* = 3) (Figure 1 and Table 1) using the DNeasy blood and tissue kit (Qiagen, Germany), following manufacturer’s instructions, and frozen at −20°C until used. Nucleic acid was previously extracted using the Canvax HigherPurity Tissue DNA Purification Kit (Canvax, Valladolid, Spain) from *Weissella* spp. recovered in Colombia (*n* = 2), and were also evaluated (Figure 1 and Table 1). The 16S rRNA gene was amplified following the method of Weisburg et al. [23] using primers 20 F: AGAGTTTGATCATGGCTCAG and 1500R: GGTTACCTTGTTACGACTT. The 25 μL reaction mixtures contained 12.5 μL of 2x DreamTaq Green PCR master mix (Thermo Fisher Scientific, USA), 0.2 μM of each primer, 2 μL of bacterial gDNA, and RNAse free water to volume. The amplification cycle consisted of 1 cycle at 95°C for 5 min, 35 cycles of denaturation at 95°C for 30 s, annealing at 50°C for 35 s, and extension at 72°C for 65 s, with a final extension step at 72°C for 10 min. PCR reactions were performed using a SimpliAmp Thermal Cycler (Thermo Fisher Scientific, Singapore) and included a nontemplate control (Milli‐Q water only). Results were visualized using a MYECL Imager (Thermo Scientific, USA) after electrophoresis of the amplified DNA in a 1.5% agarose gel containing 1% SYBR Safe DNA gel stain (Thermo Fisher Scientific, USA).

Products were purified using the QIAquick PCR purification Kit (Qiagen, USA) following the manufacturer’s protocol and sequenced, using both forward and reverse primers for each sample, by GENEWIZ (San Francisco, CA, USA). Identity and homology percentages of the different sequences were determined using the nucleotide basic local alignment search tool (blastn) analysis available from the National Centre for Biotechnology Information (NCBI) (https://blast.ncbi.nlm.nih.gov/Blast.cgi?PROGRAM=blastn&PAGE_TYPE=BlastSearch&LINK_LOC=blasthome).

### 2.6. Whole‐Genome Sequencing and AutoMLST Analysis

Whole bacterial genome sequencing of isolates listed in Table 1 was performed by Plasmidsaurus (Eugene, OR) using Oxford Nanopore Technology (ONT) and their standard (<7 Mb)s bacterial genome sequencing service level. Genome assembly and annotation were conducted using the standard Plasmidsarus pipeline (https://plasmidsaurus.com/bacteria_sequencing). Sequencing libraries were constructed using v14 library prep chemistry and sequenced on R10.4.1 flow cells. The assembly was generated by first removing low‐quality reads (5%) and downsampling the remaining reads to 25 Mb using Filtlong v0.2. A rough sketch of the assembly was created using Miniasm v0.3, and reads were further reduced to~100x coverage. Reads were then assembled using Flye v2.9.1 with parameters selected for high‐quality reads, and the resulting assembly annotated using Bakta v1.6.1 [24]. Sequencing data were submitted to NCBI under BioProject PRJNA1331700 and Biosamples SAMN51619769‐SAMN51619777. Genomes were deposited in the whole‐genome shotgun database (WGS; Table S1). The genomes were reannotated using the automated Prokaryotic Genome Annotation Pipeline (PGAP). multilocus sequence typing (MLST) analysis was performed using the AutoMLST pipeline set to de novo mode and default parameters [25]. Comparator genomes were selected automatically based on Average nucleotide identity (ANI) estimations made to the input genomes. A set of 84 single‐copy genes (Table S2) was selected and concatenated for tree building (84,032 bp compared). Model finder was used to find the optimal model for building, and bootstrap analysis (1000 replicates) was performed by IQ‐Tree 2 [26]. The Interactive Tree of Life Tool V7 (iTOL) was used to visualize the phylogenetic tree (https://itol.embl.de/) [27]. Whole‐genome multiple alignment was performed in CLC Genomics Workbench v25.0.2 (QIAGEN) using the whole‐genome alignment tool. ANI comparisons between all trout isolates and *W. ceti* from the beaked whale using default settings.

### 2.7. Genome Evaluation for Presence of Antimicrobial Resistance Genes

The nucleotide sequences of nine clinical isolates (R24462, R24464, R24466, R244610, R24492, R24493, R25071, R25075, and R250725) as well as the nucleotide sequences of two *W. tructae* reference genomes were downloaded from GenBank NCBI (Accession Numbers NZ‐CP007588.1 and NZ_CP075544.1) were analyzed via the AMRFinderPlus v4.0.23 tool (database Version 2025–07−16.1) [28], the Comprehensive Antibiotic Resistance Database (CARD—https://card.mcmaster.ca/analyze/rgi) Resistance Gene Identifier (RGI) software (RGI v.6.0.5, CARD v.4.0.1) [29], and the ResFinder v4.7.2 tool [30] to assess the presence of AMR genes. Default thresholds for AMRFinderPlus were applied using a minimum 90% identity threshold and a default 50% minimum coverage threshold. Default settings were applied for the CARD RGI tool. For ResFinder, parameters of a minimum threshold for ID of 90% and a minimum length of 60% were applied.

### 2.8. Western Blot

For the production of rabbit antisera, *W. tructae* isolate NC36 was grown in Man Rogosa and Sharpe (MRS) broth (BD, Franklin Lakes, NJ) for 24 h at 30°C in sealed bottles. Cell concentrations were estimated by direct plate counting before inactivation in 0.8% formalin. Cell concentrations were then adjusted to 1.0 × 10^9^ cells/mL, tested for sterility, and sent to Pacific Immunology, San Diego, CA (www.pacificimmunology.com) for production of polyclonal antisera in male New Zealand White rabbits weighting ~10 pounds. Antiserum was produced using the company’s custom polyclonal antibody production package under IACUC protocol # 2.18.13 ref. SOP‐1. The following inoculation schedule was utilized rabbits were primed by injection with 0.5 mL of cells mixed 1:1 with complete Freund’s adjuvant, followed by an injection with 0.5 mL of cells mixed 1:1 with incomplete Freund’s adjuvant 25, 46, and 74 days later. The serum was then collected 105 days after the prime immunization. For Western blot analysis, bacterial whole‐cell extracts were prepared by pelleting 10 mL of cells grown under conditions described above, washed twice in 2 mL of 50 mM tris (pH 8.0), and resuspended in 50 mM tris pH 8.0 containing 1 mg/mL hen white lysozyme (Sigma–Aldrich, Burlington, MA) normalized to cell number estimated by optical density. Lysozyme treatment was carried out for 1 h at 37°C, after which an equal volume of 2× SDS‐PAGE loading buffer (Bio‐Rad, Hercules, CA) was added, and the preparations were boiled for 15 min. Samples were then split, and half of each lysate was treated with 200 μg/mL proteinase K (Promega, Madison, WI) for 1 h at 65°C to digest protein antigens. Preparations (10 μL per lane) were resolved by SDS‐PAGE with an 8%–16% Criterion TGS precast gradient gel, as directed by the manufacturer (Bio‐Rad, Hercules, CA). Samples were electroblotted onto nitrocellulose membranes for 1 h at 100 V. Membranes were blocked with EveryBlot Blocking Buffer (Bio‐Rad, Hercules, CA) for 1 h at room temperature, washed once in phosphate buffer saline (PBS), and incubated overnight at 4°C with antiserum diluted 1:4,000 in blocking buffer. After three washes in PBS with 1% Tween‐20 (PBST) for 5 min each, membranes were incubated for 1 h at room temperature with goat anti‐rabbit HRP‐conjugated secondary antibody (1:4,000; Bio‐Rad, product #170−8236). Following three additional PBST washes, rabbit antibodies were detected using the Immun‐Blot Opti‐4CN detection kit (Bio‐Rad, Hercules, CA) according to the manufacturer’s instructions. All antibody dilutions were prepared in a blocking buffer. Washes and secondary antibody incubations were performed at room temperature using a Bandmate Automated Western Blot Processor (Invitrogen, Waltham, MA). The primary antibody concentration was optimized by probing identical blots with serial dilutions to achieve strong specific bands with minimal background. No signal was detected when using the preimmune serum.

### 2.9. Laboratory‐Controlled Challenges

Prior to in vivo challenges, all methods were reviewed and approved by the University of California—Davis Institutional Animal Care and Use Committee (Protocol# 23638). Rainbow trout (~9 g each) were obtained from sources with no known history of weissellosis and maintained at the Center for Aquatic Biology and Aquaculture at the University of California, Davis. Prior to challenge experiments, a subset of fish (*n* = 10) was euthanized by immersion in 500 mg L^−1^ buffered tricaine methanesulfonate (MS‐222; Syndel, Ferndale, WA, USA) for confirmation of disease‐free status by gross observation for disease signs and brain and posterior kidney culture for pathogenic bacteria on tryptic soy agar with 5% sheep blood (Biological Media Services, Davis, CA) incubated at 25°C for 7 days.

Fish (*n* = 20 per tank) were housed in 19 L flow‐through tanks supplied with supplemental aeration and fresh well water at a flow rate of 10 L m^−1^ in a biosecurity level 2 facility approved by the California Department of Fish and Wildlife to conduct laboratory‐controlled infectious disease challenges. Water temperature was monitored daily and maintained at either 18 ± 1 or 13 ± 1°C. Dissolved oxygen in all systems was measured weekly and kept at >9 mg L^−1^. Fish were fed a commercial pelleted diet (Skretting, USA) at 2% of body weight per day and acclimated to tanks for at least 1 week prior to the bacterial challenge.

The *W. tructae* isolate used in this study, R24462, was recovered from a diseased rainbow trout at a Costa Rican hatchery in 2024, revived from frozen stocks on tryptic soy agar with 5% sheep blood (BSA; Biological Media Services, Davis CA), and incubated at 28°C for 24 h. Bacteria were suspended in 5 mL of 1x PBS to match a 0.5 McFarland turbidity standard (~10^8^ CFU mL^−1^) and enumerated using a 6 x 6 drop plate method, with 10 μL of each 10‐fold dilution plated on blood agar and incubated at 30°C for 48 h [31]. Fish were challenged by ICe injection with 3.5 x 10^7^ CFU/fish.

Eight treatments were investigated (Table 2). Each treatment consisted of triplicate tanks, each holding 10 shedders and 10 cohabitant fish at the different temperatures. Prior to injection, rainbow trout “shedders” acclimatized to either 13 or 18°C for 2 weeks were anesthetized under supplemental aeration with 50 mg L^−1^ buffered MS‐222 and injected with 0.1 mL of sterile PBS (negative controls) or their respective bacterial suspension by ventral ICe injection slightly dorsal and cranial to the anus. Following injection, the adipose fin was removed from shedders, and fish were returned to their experimental tanks. Cohabitant fish were exposed to bacteria through horizontal transmission via the ICe‐challenged shedders.

**Table 2:** Treatments of rainbow trout used in laboratory‐controlled challenges.

Treatment	Exposure route	*Weissella tructae*	Temperature (°C)
A.	Intracelomic injection	Yes	18 ± 1
B.	Intracelomic injection	Yes	13 ± 1
C.	Intracelomic injection	No	18 ± 1
D.	Intracelomic injection	No	13 ± 1
E.	Cohabitation	Yes	18 ± 1
F.	Cohabitation	Yes	13 ± 1
G.	Cohabitation	No	18 ± 1
H.	Cohabitation	No	13 ± 1

Fish were monitored twice daily for 21 days postinjection for the onset of clinical signs, morbidity, and mortality. Moribund fish were defined as having either one severe clinical sign, two or more clinical signs at one time, or one clinical sign lasting more than 24 h. Moribund trout were euthanized following AVMA guidelines for euthanasia [32].

Moribund, “fresh‐mortalities,” and survivors were collected to assess bacterial persistence. Aseptic swabs of brains were inoculated onto tryptic soy agar supplemented with 5% sheep blood and incubated at 25°C for 24–48 h. Isolation of GP, catalase‐negative, and cytochrome oxidase‐negative cocci was interpreted as positive recovery of *W. tructae*. For histopathologic evaluation, a subset of moribund fish was fixed whole in 10% NBF with their celomic cavities incised. The whole bodies were demineralized for 24 h in Kristensen’s solution and serially transected at ~2 mm intervals through the head and trunk/celomic regions into histology cassettes. Tissue processing and staining were done as previously described.

Survival curve analyses were performed using GraphPad Prism (Version 8.3.0, GraphPad Software, La Jolla, CA, USA). Statistical significance was determined by log‐rank (Mantel–Cox) and Gehan–Breslow–Wilcoxon tests.

## 3. Results

### 3.1. Clinical and Histological Findings of Naturally Infected Fish

Mortalities reached >60% in some systems, affecting most life stages, but broodstock and fish >80 g were disproportionally affected. Water temperatures exceeding 15°C appeared to facilitate the outbreaks in most cases (Table 1). Per history provided by aquaculturists, other water quality parameters (dissolved oxygen, ammonia, nitrate, nitrites, alkalinity, and hardness) were within normal limits for rainbow trout [33]. Infected fish were lethargic and swam erratically, with most exhibiting bilateral exophthalmia, corneal opacity, ocular and perianal hemorrhages, and melanosis (Figure 2). Internal findings included ascites, splenomegaly, gut congestion, and hemorrhages in the swim bladder, brain, liver, and skeletal muscle.

**Figure 2:**
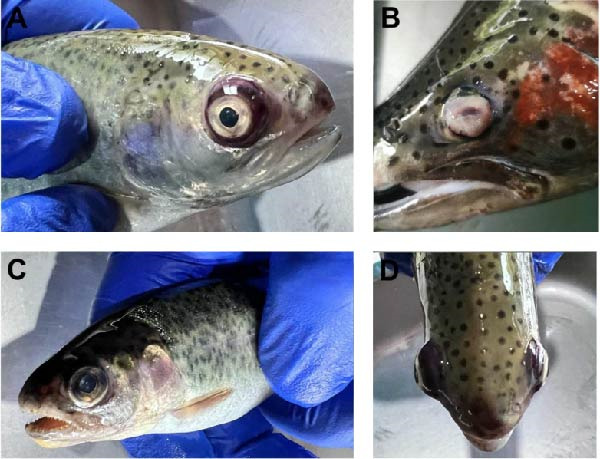
Gross changes in naturally infected rainbow trout with *Weissella tructae* in Costa Rica. Moribund fish presented with exophthalmia, periocular hemorrhages (A–D), and melanosis (B–D). (A) Fish sampled from San Gerardo de Dota, Costa Rica. (B) Fish sampled from Aserri, Costa Rica. (C) Fish sampled from San Gerardo de Dota, Costa Rica. (D) Fish sampled from Cedral, Perez Zeledón, Costa Rica.

Histopathologic findings were consistent with published descriptions of *Weissella* infection in rainbow trout, including periocular hemorrhage and inflammation, hemorrhagic coelomitis, aerocystitis, mural gastroenteritis, and subjectively splenomegaly (Figure 3). Best visualized using the tissue Gram stain, free and phagocytized GP coccobacilli were often individualized and inconspicuous but never present in more than small numbers (Figure 3B,D). Microscopic changes, as well as bacterial numbers, were the greatest in retroperitoneal connective tissues surrounding the swim bladder. Lesions included hemorrhage, edema, and mixed inflammatory cell infiltrates dominated by neutrophils, with small numbers of macrophages and scattered lymphocytes. Milder changes were extended throughout the swim bladder and intestinal walls, as well as the periocular connective tissues. Changes and bacterial numbers in the spleen were minimal (Figure 3).

**Figure 3:**
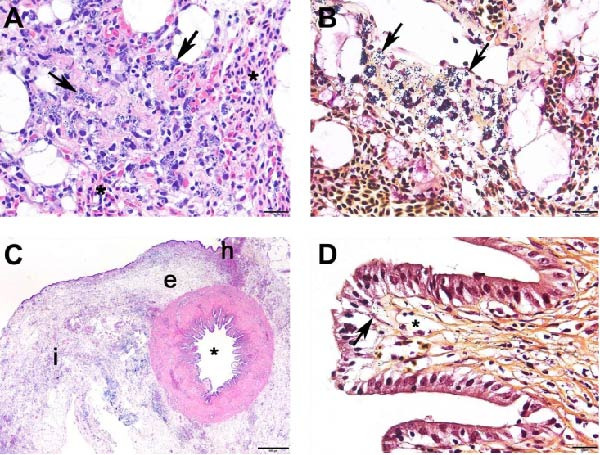
Histopathologic findings associated with the swim bladder of a rainbow trout naturally infected with *Weissella tructae*. (A) Free and phagocytized coccobacilli (arrows) within a hemorrhagic areas (asterisks) of retroperitoneal adventitia adjacent to the swim bladder. (H&E stain, bar = 20 µm). (B) In a section corresponding to (A), bacteria stained strongly Gram‐positive (arrows) using a tissue Gram stain (Brown and Hopps stain, bar = 20 µm). (C) Microscopic changes and bacterial numbers were greatest in retroperitoneal connective tissues surrounding the swim bladder (asterisk). Lesions included hemorrhage (h), edema (e) and mixed inflammatory cell infiltrates (i) dominated by neutrophils. (H&E stain, bar = 500 µm). (D) Changes in the highly contracted wall of the swim bladder were minimal. The image highlights submucosal edema (asterisk) and individualized, inconspicuous bacteria (arrow) best visualized with a Gram stain. (Brown and Hopps stain, bar = 20 µm).

### 3.2. Bacterial Identification, Morphological, Biochemical, and Antimicrobial Susceptibility Characteristics

Cultured on blood agar, circular, white, opaque colonies were 0.5–1.0 mm in diameter, with regular margins and slightly convex elevations after 24–48 h of incubation at 25°C (Figure S1A and B). Distinct α‐hemolytic activity was evidenced by a greenish halo surrounding the colonies. Microscopically, the cells were GP, nonmotile, and non‐spore‐forming, appearing as short rods or cocci. Biochemical testing revealed negative catalase and oxidase activity (Figure S1A and B), consistent with the characteristics of the genus *Weissella*. Using MALDI‐TOF MS, isolates were assigned to the genus *Weissella*, supporting the phenotypic identification. In contrast, the Vitek 2 system failed to identify isolates even to the genus, yielding only a single positive substrate reaction for arginine dihydrolase 1.

The minimal inhibitory concentration values of different antimicrobials against the *W. tructae* isolates are presented in Table 3. The *E. coli* reference strain ATCC 25922 profiles were within ranges established by CLSI [21].

**Table 3:** Minimal inhibitory concentrations (µg/mL) of 19 antimicrobials against *Weissella tructae* isolates recovered from Costa Rica.

Antimicrobial	R24462	R24464	R24466	R244610	*E. coli* ATCC 25922	*E. coli* ATCC 25922 ranges per CLSI [21]
Enrofloxacin	2	>2	2	>2	<or = 0.12	0.008–0.03
Ceftiofur	>4	>4	>4	>4	0.5	NA
Neomycin	<or = 2	<or = 2	<or = 2	<or = 2	<or = 2	NA
Erythromycin	0.25	0.25	<or = 0.12	<or = 0.12	>4	NA
Oxytetracycline	8	8	8	8	1	0.5–2
Tetracycline	8	8	8	8	1	NA
Amoxicillin	<or = 0.25	<or = 0.25	<or = 0.25	<or = 0.25	8	NA
Spectinomycin	64	64	64	64	16	NA
Sulphadimethoxine	>256	>256	>256	>256	>256	NA
Trim/sulfa	2/38	>2/38	>2/38	>2/38	<or = 0.5/9.5	0.03/0.06−0.25/4.8
Florfenicol	2	4	2	4	4	4–16
Sulfathiazol	>256	>256	>256	>256	128	NA
Penicillin	2	4	4	4	>8	NA
Streptomycin	32	32	32	32	<or = 8	NA
Novobiocin	>4	>4	>4	>4	>4	NA
Tylocin tartrate	<or = 2.5	<or = 2.5	<or = 2.5	<or = 2.5	>20	NA
Clindamycine	<or = 0.5	<or = 0.5	<or = 0.5	<or = 0.5	>4	NA


Abbreviation: NA, not available.

### 3.3. Molecular Identification

Amplicons from the targeted 16S rRNA PCR were of the anticipated size (~1500 bp). Consensus sequences of these amplicons were 100% identical to that of a *W. tructae* 16S ribosomal RNA gene partial sequence in the NCBI nr database based on query results using the blastn. This confirmed the isolates as *W. tructae* and prompted complete genome sequencing efforts.

### 3.4. Multilocus Sequence Analysis

Phylogenetic analysis was performed based on the comparison of 84 concatenated genes found in all analyzed genomes (84,032 aligned positions) using the web‐based AutoMLST pipeline (https://automlst.ziemertlab.com) (Table S2). The resulting phylogenetic tree indicates that *W. tructae* isolates from Colombia, Peru, and Costa Rica all cluster closely with previously identified isolates from Mexico, Brazil, and the United States (Figure 4A). Alignment of the 84 genes from just the *W. tructae* isolates further establishes the high level of genetic similarity between these isolates, with most (11/14) showing a 100% identity over the 84,032 bp region (Figure 4B). Colombian isolates R24493 and R24492 were identical to each other and differed from the consensus at four positions. In contrast, the Mexican isolate (W1) differed at a single position. Whole‐genome ANI analysis was performed using coding and noncoding nucleotides. This analysis supported observations from the MLST, highlighting the similarity of R24492 and R24493. These isolates were >99.99% identical across the whole genome (Figure 5). The percent ANI between the new isolates of *W. tructae* presented here was >99.99%. The greatest difference in ANI was observed between isolate WS74 and isolate R24462, which were 99.82% identical. The shared identity with the nearest *Weissella* neighbor, *W. ceti*, ranged from 85.72% to 85.93%.

**Figure 4:**
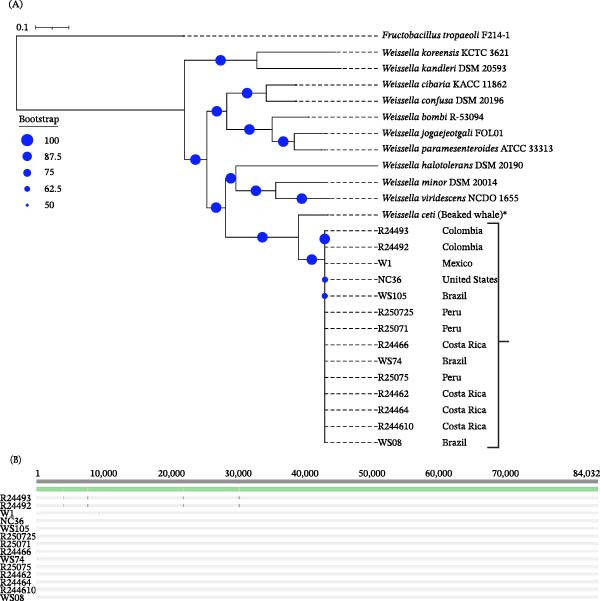
(A) Maximum likelihood phylogenetic tree of *W. tructae* isolates in relation to other members of the *Weissella* genus, and the closest described neighbors of the *Weissella* genus *Weissella* neighbor *W. ceti* (beaked whale; indicated by an  ^∗^) (Table S3). The tree was computed based on 84 single‐copy genes totaling 84,032 aligned positions using the AutoMLST tool (see Section 2 for details) and visualized with iTOL V7. *W. tructae* isolates indicated by bracket. (B) Multiple alignment of the 84,032 bp region from the *W. tructae* isolates examined. Single nucleotide polymorphisms are represented by vertical lines.

**Figure 5:**
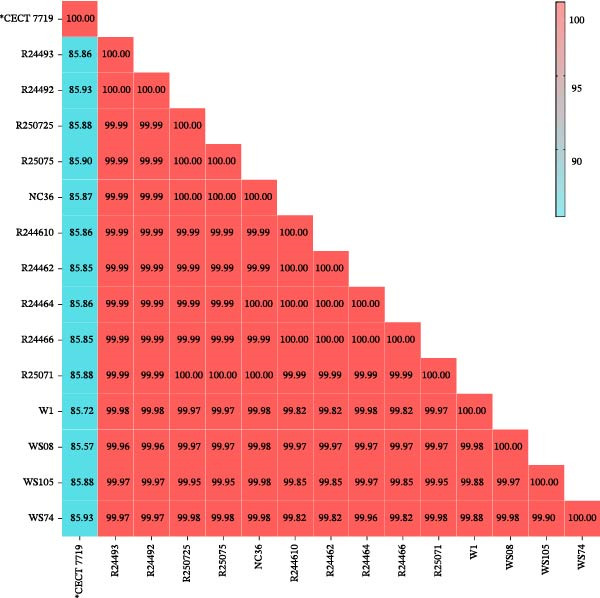
Average nucleotide identity matrix of *W. tructae* genomes and the closest *Weissella* neighbor *W. ceti* (beaked whale; indicated by an asterisk) (Table S3). Percent pairwise identity is denoted.

### 3.5. Antimicrobial Resistance Gene Detection

No AMR genes were detected from any of the 10 isolates using the ResFinder and AMRFinder tools. The CARD RGI software identified two glycopeptide antibiotic AMR genes (*vanY* and *vanT*) present in all 10 isolates.

### 3.6. Western Blot

Whole‐cell protein profiles from US isolate NC36 and those from Costa Rica were identical when analyzed by SDS‐PAGE (Figure 6A). Whole‐cell lysates were also subjected to western blotting using polyclonal rabbit antisera raised against NC36 whole cells, revealing 11 major antigenic bands shared among all isolates, with no apparent differences in banding pattern or intensity detected (Figure 6B). Blotting of lysates after pretreatment with proteinase K demonstrated antigen sensitivity to protease, confirming that they consisted of protein. This analysis also allowed the detection of nonprotein antigens, which were also similar in both migration and signal intensity (Figure 6B). These results suggest that Costa Rican isolates are antigenically similar to North Carolina isolate NC36.

**Figure 6:**
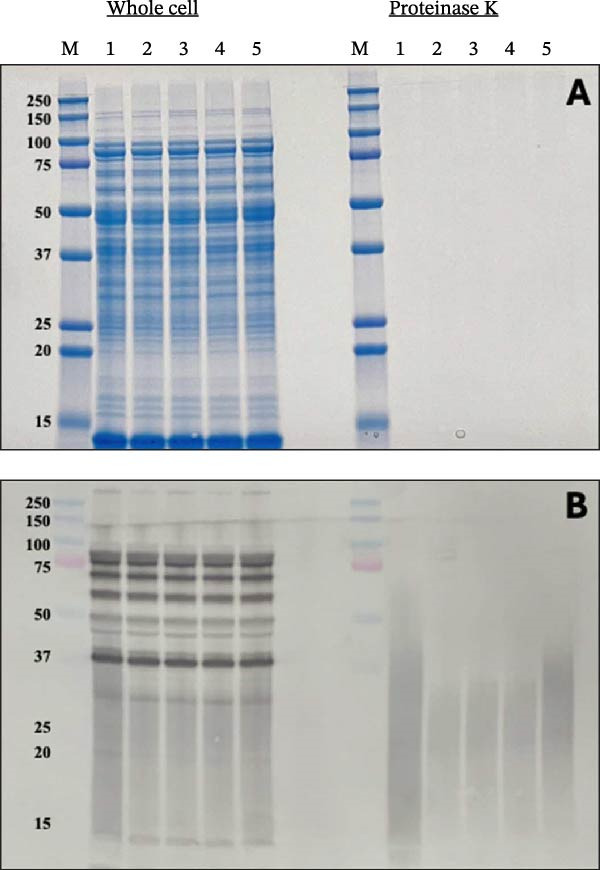
United States and Costa Rican isolates are antigenically homogeneous. (A) Coomassie blue‐stained SDS‐PAGE analysis whole‐cell lysates of *W. tructae* isolates. Samples on the right side of gel were pretreated with proteinase K. (B) Detection of shared antigens by western blot analysis using antiserum prepared in rabbits by immunization with whole cells of isolate NC36. The following isolates were analyzed: lane 1, NC36 (North Carolina); lane 2, R24462 (Costa Rica); lane 3, R24464 (Costa Rica); lane 4, R24466 (Costa Rica); lane 5, R244610 (Costa Rica).

### 3.7. Laboratory‐Controlled Challenges

Mortality was impacted by the environmental temperature and challenge method. Trout challenged with *W. tructae* at 18°C via ICe injection experienced 96.7% mortality (29/30 fish) in the 21‐day trial (Figure 7). At 18°C, the first mortalities occurred on day 2 postinjection. In contrast, fish challenged at 13°C experienced significantly lower mortality (40% mortality; 16/40 fish) (*p* < 0.001), with the first death on day 6 post‐ICe injection (Figure 7). Trout cohabitated with *W. tructae*‐infected fish at 18°C experienced 16.7% (5/30 fish) mortality in the 21‐day challenge period (Figure 7), with first mortalities occurring on day 18. In contrast, cohabitant fish challenged at 13°C experienced no mortality (*p* = 0.02). No mortalities or signs of disease occurred in control tanks at either temperature (data not shown).

**Figure 7:**
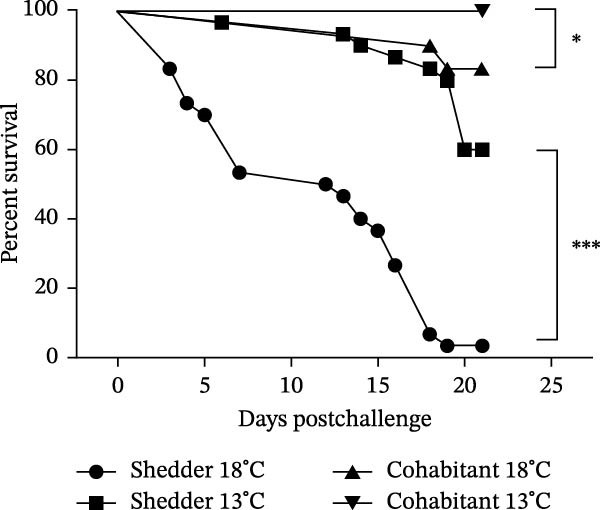
Survival curves in rainbow trout fingerlings challenged with *Weissella tructae* from Costa Rica. Trout were challenged by either intracelomic injection with ~10^7^ CFU/fish or by cohabitation at 13 or 18°C. Each treatment consisted of triplicate tanks, each containing 10 fish.  ^∗^ *p* < 0.05 and  ^∗∗∗^ *p* < 0.0001.

Clinical signs and external gross changes exhibited by moribund and dead trout included lethargy, inappetence, cutaneous melanosis, unilateral and bilateral exophthalmia, and hyphema (Figure 8). Presumptive *W. tructae* was reisolated from the brains of all sampled mortalities. *Weissella tructae* was recovered from the brains of 1/1 and 1/25 surviving shedder and cohabitant fish challenged at 18°C, respectively, and from 16/18 and 1/30 surviving shedder and cohabitant fish challenged at 13°C, respectively.

**Figure 8:**
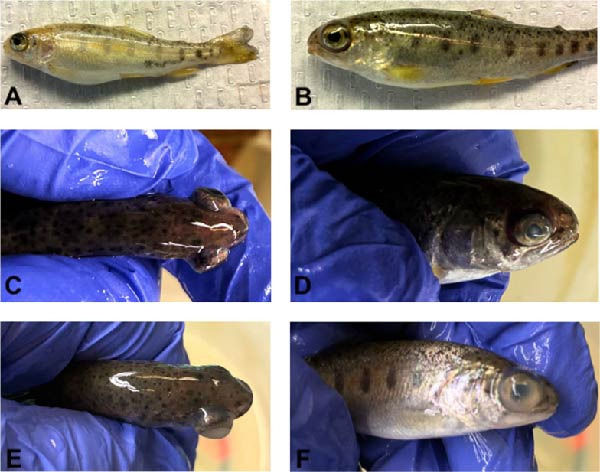
Gross changes in rainbow trout laboratory challenged with *Weissella tructae* recovered from Costa Rica. Moribund fish evaluated 3 (A, B), 7 (C, D) and 20 (E, F) days post challenge exhibited celomic distention, exophthalmia, buphthalmia, periocular hemorrhages, and melanosis. These subparts (A–D) are moribund fish exposed at 18°C. These subparts (E, F) are fish exposed at 13°C.

Histopathologic findings among the naturally infected and laboratory‐challenged rainbow trout were microscopically similar, varying primarily in the severity of lesions and numbers of visible bacteria (Figures 9 and 10). However, changes were evident between the injected “shedder” and cohabitated fish and at the two temperatures (Figure 9). All fish examined were in good physical condition, evidenced by robust musculature, abundant celomic adipose, and often the presence of ingesta within gastrointestinal tracts. In the six 18°C shedder group fish, the periocular adipose was edematous with large numbers of free and phagocytized bacteria. Inflammatory infiltrates were generally mild and dominated by neutrophils. Changes were widely distributed in the periocular adipose but tended to localize in perivascular and other adventitial sites such as those surrounding the vestibular membranous labyrinth. Bacterial numbers and inflammatory changes were most pronounced in association with the optic nerve sheaths, particularly near the optic chiasm, and occasionally extended into the ventral meninges. Interstitial areas of adjacent skeletal muscle were often similarly affected, accompanied by necrosis of individual myofibers (Figure 9A–C). In severe cases, changes extended widely into the isthmus and pericardial muscle and cranially between the nasal sacs. Pathologic changes only involved the inner ocular structures of two fish, characterized by retinal detachment, subretinal hemorrhage, and free and phagocytized bacteria, and were most severe near the optic discs (Figure 9D). Rare bacteria and neutrophils infiltrated the anterior chamber of one fish. Additional areas less commonly affected included the epicardium and pericardial cavity, pseudobranchs, retroperitoneal connective tissue ventral to the swim bladder, and adventitial tissues surrounding the cranial lateral line canals and dermis. Internally, the spleens of the ICe‐injected fish were enlarged by marked congestion. Periarteriolar sheaths were dominated by macrophages with cytoplasm distended by large numbers of bacteria (Figure 9E). Renal sinusoids were variably dilated and congested with only infrequently phagocytized bacteria. Necrosis in both tissues was minimal and limited to scattered individual cells. Renal hematopoietic areas were dominated by large immature cells with high nuclear‐to‐cytoplasmic ratios and frequent mitoses.

**Figure 9:**
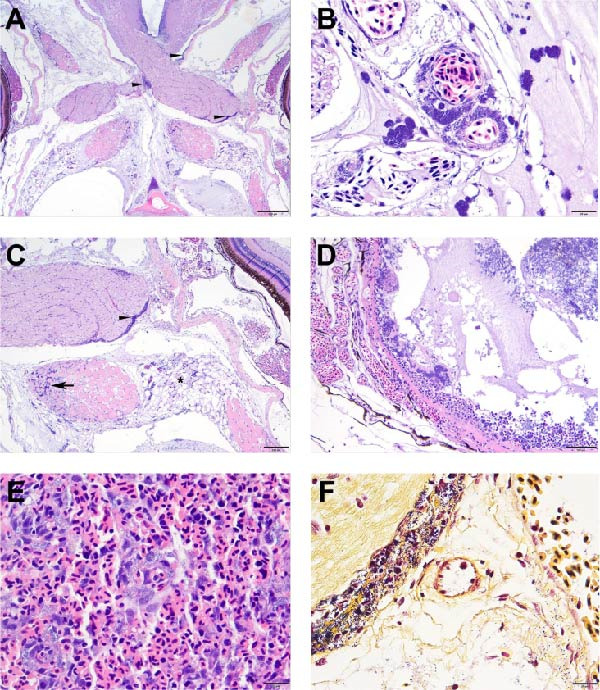
Histopathologic findings in “shedder” rainbow trout *Oncorhynchus mykiss* challenged by intracelomic injection with *Weissella tructae* and maintained at 18°C. (A) Cephalic cross section at the level of the eyes. Basophilic foci (arrowheads) represent masses of bacteria widely distributed throughout the head. (H&E stain, bar = 500 µm). (B) Bacteria were frequently localized to areas of abundant connective tissue, such as perivascular walls. (H&E stain, bar = 20 µm) (C) Bacteria concentrated within the optic nerve sheath (arrowhead), interstitial areas of skeletal muscle (arrow), and periocular adipose (asterisk). (H&E stain, bar = 200 µm). (D) Eye severely affected by retinal detachment creating a space partially filled by proteinaceous fluid, large numbers of bacteria, and inflammatory cells dominated by neutrophils. (H&E stain, bar = 100 µm). (E) Spleen with periarterial sheaths containing macrophages with cytoplasm distended by bacteria. (H&E stain, bar = 20 µm). (F) Gram‐stained section of optic nerve picture in (C) demonstrating large numbers of pleomorphic Gram‐positive bacteria. (Brown and Hopps stain, 20 µm).

**Figure 10:**
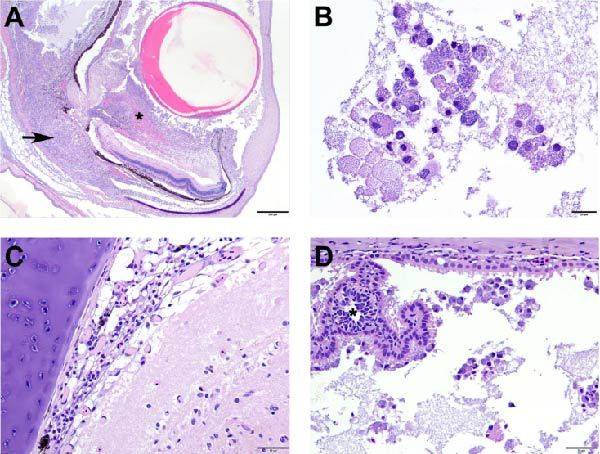
Histopathologic findings in rainbow trout *Oncorhynchus mykiss* cohabitated with *Weissella tructae* injected shedders and maintained at 18°C. (A) Panophthalmitis with intense subretinal inflammatory infiltrates (arrowhead), involvement of the intraocular chambers (asterisk), and dissemination to periocular adipose tissue. (H&E stain, bar = 500 µm). (B) Neutrophils and phagocytized bacteria within the anterior chamber of the eye. (H&E stain, bar = 20 µm). (C) Subacute to chronic meningitis dominated by lymphocytes with fewer neutrophils. Cranial cartilage is to the left and optic lobe of the brain to the right. (H&E stain, bar = 50 µm). (D) Ventriculitis, with inflammatory cells and bacteria in the extension of the third ventricle surrounding the pineal stalk (asterisk). (H&E stain, bar = 50 µm).

In contrast to the injected shedders, changes within the eyes of three 18°C cohabitating trout were severe (Figure 10A), while those in the periocular tissues were relatively mild. Subretinal lesions were similar to those above but more intense and chronic in nature, including greater numbers of lymphocytes and areas of fibroplasia. In addition, ocular chambers were infiltrated by moderate to large numbers of neutrophils and contained predominantly intracellular bacteria (Figure 10B). Inner retinal nerve fiber layers were degenerate and infiltrated by smaller numbers of inflammatory cells and bacteria. Evidence of meningitis in the brains of these fish was more widespread than that in the shedders (Figure 10C). Small numbers of neutrophils and macrophages with cytoplasm distended by bacteria were present within their third ventricles and extended around the pineal stalks of two fish (Figure 10D). The involvement of adventitial connective tissue surrounding the nasal sacs was observed in one fish but did not spread to the olfactory epithelium. Mild subacute coelomitis was present in two trout, but the splenic, renal, and hematopoietic changes described above were not observed.

Findings in the four 13°C shedder trout were generally similar to but less consistent and, with the exception of one fish, less severe than those in the corresponding 18°C group. Intraocular changes were mild, while periocular and intracephalic changes were minimal in the three fish. In the fourth, the findings more closely resembled those in the 18°C shedders. No bacteria were observed in the spleens of two fish, with a few and large numbers in the spleens of one each.

## 4. Discussion

This is the first study to describe *W. tructae* outbreaks in farmed rainbow trout in Costa Rica and Central America. The *W. tructae* isolates recovered from Costa Rica showed considerable genetic homogeneity with those recovered from outbreaks in Colombia and Peru, as well as with published genomes from isolates from Mexico, Brazil, and North Carolina, USA. Comparison of antigenic profiles among *W. tructae* from Costa Rica and North Carolina confirmed that the isolates are also antigenically homogenous. The exceptional homogeneity of the isolates suggests that the outbreaks may have originated from a common source, although further work is needed to fully understand the phylogeography of this pathogen.

Results from the present study and previous reports, as well as observations from seasonal outbreaks on fish farms around the world, demonstrate that warmer water is associated with a higher risk of weissellosis [11, 16, 34]. Mortality of ~100% was achieved in rainbow trout at 18°C at a dose that only caused 40% mortality at 13°C (Figure 7). These results are comparable to findings from Costa et al. [34], who tested a single dose of *W. tructae* in trout (10^7^ CFU fish^−1^ ICe) and produced 100% mortality in naive fish at similar temperatures.

While a clear impact on morbidity and mortality was observed between fish exposed at the two temperatures, elucidating mechanisms by which temperature influences disease progression is difficult given that temperature effects conflate factors impacting pathogen virulence, host immunity, and water quality. From the microbial perspective, temperature can induce differential expression of growth and virulence genes [36], although no studies have investigated the relationship between temperature and virulence for *W. tructae*.

In *Edwardsiella piscicida*, the PhoP–PhoQ two‐component regulatory system mediates temperature sensing and controls the expression of both the type III and type VI secretion systems (T3SS and T6SS), which are downregulated at 37°C relative to 25°C [35, 36]. Similarly, when *E. piscicida* isolates were cultured at 37°C, comparative proteomic analysis by mass spectrometry revealed a marked downregulation of key virulence‐associated proteins, including components of the type VI secretion system and flagellar proteins, relative to growth at 27 and 17 °C [37]. In GP bacteria like *Streptococcus agalactiae*, elevated temperature upregulated the expression of genes encoding key virulence factors, particularly Bsp, PcsB, and the CAMP factor. These proteins contribute to pathogenesis by promoting adhesion of group B streptococci to the host cell surface, stimulating bacterial cell division, and inducing host cell death through pore formation in host cell membranes [38].

Warmer temperatures can also provide a more favorable environment for *W. tructae* replication, which is faster at 18°C than at 13°C (Soto et al. unpublished). In addition, 16/18 (89%) fish surviving the 21‐day ICe challenge at 13°C yielded positive cultures from their brains. Although the influence of temperature on bacterial persistence needs to be explored further, the findings suggest the possibility of a carrier state developing at temperatures under which trout are cultured. Bacterial persistence in surviving fish following disease outbreaks is common in aquaculture systems and can serve as a reservoir of infection for naïve fish introduced into the system, thereby contributing to recurrent disease outbreaks. This phenomenon has been documented in francisellosis, mycobacteriosis, and other chronic or recurring bacterial diseases affecting a wide range of cultured fish species [39, 40].

Although conclusions should not be made based on the small sample sizes, differences were observed histologically between surviving injection‐ and cohabitation‐challenged fish and at the two temperatures. Despite severe lesions, most fish remained in good body condition, and many continued to feed, evidenced by abundant ingesta in their gastrointestinal tracts. Consistent with earlier reports, *W. tructae* had a predilection for the eyes and periocular tissues, regardless of temperature or infection route. However, using the natural cohabitation route, lesions were confined primarily to the eyes and cranial soft tissues, while most fish injected intracelomically also had large numbers of phagocytized bacteria in their spleens and occasionally in their kidneys. In contrast, injected fish tended to have less central nervous system involvement. This was observed particularly at 18°C, where intraocular changes predominated in cohabitated fish but tended to remain periocular in injected fish. Regardless of the challenge route, bacteria were primarily associated with adventitial connective tissue composing vascular walls and nerve sheaths, as well as that surrounding the lateral line, membranous labyrinth, and swim bladder, and rarely infiltrated deeper into functional structures. The almost complete absence of changes associated with the nasal sacs suggests that the olfactory epithelium may not serve as a portal of entry for the bacterium. Infrequently, there was spread to adjacent muscles in the head. Although fewer fish were examined, findings in the 13°C fish were similar but less consistent and often less severe.

Temperature can also exert profound effects on host immunity, which is maximized when environmental conditions are ideal for a given species. For rainbow trout, temperature optima range between 13–16°C, above which all outbreaks of weissellosis published to date have occurred [11, 16, 34]. Immunosuppression and other negative physiological effects induced by temperature stress increase susceptibility to infection despite innate and adaptive mechanisms by fish to counter such adverse consequences [41–43]. Elevated temperatures can also impact fish immune systems indirectly by affecting other water quality parameters, such as dissolved oxygen and unionized (toxic) ammonia, which create further stress. Although dissolved oxygen levels during the laboratory challenges remained optimal for trout (>9 mg L^−1^), elevated temperatures could have influenced mortalities during the natural outbreaks by lowering dissolved oxygen levels and increasing their vulnerability to pathogens [41–45].

Currently, few tools are available to prevent and control outbreaks of weissellosis. Welch and Good [13] reported relative percent survivals (RPS) of 87.5% and 85% at 38 and 72 days, respectively, following ICe administration of a formalin‐inactivated whole‐cell vaccine derived from US *Weissella* isolates. Costa et al. [34] reported similar results using a formalin‐killed bacterin based on Brazilian isolates, and as a result, Brazil, Colombia, Peru, and other countries have adopted this immunization method. However, due to international regulations and the lack of a regionally available commercial product, countries such as Costa Rica still lack an approved vaccine. Moving forward, producers advocate for the development of oral or immersion mucosal vaccines that would not require individual injection, particularly in fry and small fingerlings.

Antimicrobial therapy remains a useful tool in aquaculture when used judiciously following a definitive diagnosis and antimicrobial susceptibility testing. However, regulations differ between countries regarding the use in fish destined for human consumption. To the author’s knowledge, reports of *W. tructae* antimicrobial susceptibilities are few, and approved breakpoints are lacking. Although broth microdilution methods were used in both studies, MICs in the current investigation differed from those of the Vietnamese isolates reported by Nhinh et al. [12]. However, it is uncertain whether the disparities reflect intrinsic differences between the isolates or unreported differences in the methods used. In the current study, an MIC of 8 μg/mL was observed for oxytetracycline under the conditions tested. Although no breakpoints are established for *W. tructae*, an MIC of 8 μg/mL would not support the use of this antibiotic as a first‐line of therapy. Recently, Heckman et al. [46] reported comparable efficacy of florfenicol‐ and erythromycin‐medicated feeds in reducing mortality and limiting the spread of lactococcosis in rainbow trout during cohabitation challenge studies. In the United States, erythromycin is approved for use as an investigational new animal drug to manage bacterial kidney disease and to reduce bacterial loads in salmonids infected with the GP pathogen *Renibacterium salmoninarum* [47]. Given the low MIC observed in the present study (≤0.25), further investigation into the in vivo efficacy of erythromycin‐medicated feed for the treatment of weissellosis is warranted. Future comparison between isolates of different geographical origins and standardization of protocols are also warranted to establish epidemiological cutoff values and propose therapeutic protocols for the industry.

## 5. Conclusions

In this study, *W. tructae* was identified as the etiological agent of disease outbreaks in cultured rainbow trout in Costa Rica. Additionally, findings indicate that *W. tructae* isolates from Latin America and the Eastern US are highly homogeneous genetically and antigenically. An important role for temperature in the pathogenicity of *W. tructae* to trout fingerlings was also evidenced by the high morbidity, mortality, and horizontal transmission that occurred in fish cultured at 18°C compared to 13°C. A consistent risk factor in affected countries is reliance on imported trout eggs and other life stages. Additionally, although some Latin American countries have implemented regulations and guidelines for egg importation, fish and fish by‐products, disease surveillance, and overall health management and biosecurity, improper importation of eggs and animals, animal smuggling, and inconsistent regulations at the regional level remain major issues for the aquaculture industry. The emergence of *W. tructae* in Latin American aquaculture is clearly demonstrated and highlights the need for continued monitoring and the establishment of better health management tools and protocols to avoid the dissemination of this pathogen.

## Funding

This study was funded by University of California‐Davis, School of Veterinary Medicine.

## Conflicts of Interest

The authors declare no conflicts of interest.

## Supporting Information

Additional supporting information can be found online in the Supporting Information section.

## Supporting information


**Supporting Information** Table S1. GenBank accession numbers of isolates used for phylogenetic comparisons. New genomes reported here are indicated with an asterisk ( ^∗^). Strains used exclusively for Figure 5 are denoted with a hash (#). Table S2. Genes used in the phylogenetic analysis of *Weissella tructae*. Table S3. GenBank accession numbers of strains used for phylogenetic comparison to *Weissella tructae* (Figures 4 and 5) Figure S1. Physiochemical characteristics of *Weissella tructae*. Colony morphology when grown overnight at 28°C in trypticase soy agar with 5% sheep blood. The opaque, white colonies were 0.5–1.0 mm in diameter (A). Evaluated against a light source, distinct α‐hemolytic activity was observed as a greenish halo surrounding colonies (B). *Weissella tructae* isolates were catalase and cytochrome oxidase‐negative (asterisk), as evidenced by a lack of bubble formation and blue color change, respectively. *Aeromonas cavieae* was used for comparison (catalase and cytochrome oxidase‐positive).

## Data Availability

The data that support the findings of this study are available from the corresponding author upon reasonable request.
