# Supplementary material for: Diagnosis of Weissellosis in Cultured Rainbow Trout in Costa Rica Further Evidence Emergence of Weissella tructae as Important Pathogen of Salmonids in Latin America
Source: Transbound Emerg Dis. 2026 Jun 15;2026:4690289. doi: 10.1155/tbed/4690289 (PMC13266906; doi:10.1155/tbed/4690289)
Supplement: Supplementary file 1 — Supporting Information Table S1. GenBank accession numbers of isolates used for phylogenetic comparisons. New genomes reported here are indicated with an asterisk ( ∗). Strains used exclusively for Figure 5 are denoted with a hash (#). Table S2. Genes used in the phylogenetic analysis of Weissella tructae. Table S3. GenBank accession numbers of strains used for phylogenetic comparison to Weissella tructae (Figures 4 and 5) Figure S1. Physiochemical characteristics of Weissella tructae. Colony morphology when grown overnight at 28°C in trypticase soy agar with 5% sheep blood. The opaque, white colonies were 0.5–1.0 mm in diameter (A). Evaluated against a light source, distinct α‐hemolytic activity was observed as a greenish halo surrounding colonies (B). Weissella tructae isolates were catalase and cytochrome oxidase‐negative (asterisk), as evidenced by a lack of bubble formation and blue color change, respectively. Aeromonas cavieae was used for comparison (catalase and cytochrome oxidase‐positive). [file TBED-2026-4690289-s001.docx]

**Supplementary Material.**

Supplementary Table 1. GenBank accession numbers of isolates used for phylogenetic comparisons. New genomes reported here are indicated with an asterisk (*). Strains used exclusively for Figure and 5 are denoted with a hash (#).

| **Isolate** | **Accession** |
| --- | --- |
| ^*^*Weissella tructae* (R244610) | JBUHYN010000001.1 |
| ^*^*Weissella tructae* (R24462) | JBUHYH010000001.1 |
| ^*^*Weissella tructae* (R24464) | JBUHYI010000001.1 |
| ^*^*Weissella tructae* (R24466) | JBUHYJ010000001.1 |
| ^*^*Weissella tructae* (R24492) | JBUBHJ010000001.1 |
| ^*^*Weissella tructae* (R24493) | JBUHYK010000001.1 |
| ^*^*Weissella tructae* (R25071) | JBUHYL010000001.1 |
| ^*^*Weissella tructae* (R250725) | JBUHYO010000001.1 |
| ^*^*Weissella tructae* (R25075) | JBUHYM010000001.1 |
| *Weissella tructae* (W1) | CP075544.1 |
| *Weissella tructae* (WS08) | CP007588.1 |
| *Weissella tructae* (WS74) | CP009223.1 |
| *Weissella tructae* (WS105) | CP009224.1 |
| *Weissella tructae* (NC36) | GCA_000320345.1 |
| *Weissella ceti CECT 7719* | CP074441 |
| *^#^Weissella koreensis* KCTC 3621 | AKGG00000000.1 |
| *^#^Weissella kandleri* DSM 20593 | NZ_JQBP00000000.1 |
| *^#^Weissella cibaria* KACC 11862 | AEKT00000000.1 |
| *^#^Weissella confusa* DSM 20196 | NZ_JQAY00000000.1 |
| *^#^Weissella bombi* R-53094 | NZ_FMAO00000000.1 |
| *^#^Weissella jogaejeotgali* FOL01 | [CP014332.1](https://www.ncbi.nlm.nih.gov/nuccore/CP014332.1/) |
| *^#^Weissella paramesenteroides* ATCC 33313 | ACKU00000000.1 |
| *^#^Weissella halotolerans* DSM 20190 | ATUU00000000.1 |
| *^#^Weissella viridescens* NCDO 1655 | CYXF00000000.1 |
| *^#^Weissella minor* DSM 20014 | NZ_JQCD00000000.1 |
| *^#^Fructobacillus pseudoficulneus* DSM 15468 | FNWS00000000.1 |

Supplementary Table 2. Genes used in the phylogenetic analysis of *Weissella tructae*.

| NCBI accession | Gene | Function |
| --- | --- | --- |
| TIGR00055 | uppS | di-trans,poly-cis-decaprenylcistransferase |
| TIGR03953 | rplD_bact | 50S ribosomal protein uL4 |
| TIGR00447 | pth | aminoacyl-tRNA hydrolase |
| TIGR00647 | DNA_bind_WhiA | DNA-binding protein WhiA |
| TIGR00150 | T6A_YjeE | tRNA threonylcarbamoyl adenosine modification protein YjeE |
| TIGR00174 | miaA | tRNA dimethylallyltransferase |
| TIGR03632 | uS11_bact | ribosomal protein uS11 |
| TIGR01632 | L11_bact | ribosomal protein uL11 |
| TIGR00409 | proS_fam_II | proline--tRNA ligase |
| TIGR00484 | EF-G | translation elongation factor G |
| TIGR00038 | efp | translation elongation factor P |
| TIGR03725 | T6A_YeaZ | tRNA threonylcarbamoyl adenosine modification protein YeaZ |
| TIGR00981 | rpsL_bact | ribosomal protein uS12 |
| TIGR00090 | rsfS_iojap_ybeB | ribosome silencing factor |
| TIGR01066 | rplM_bact | ribosomal protein uL13 |
| TIGR01169 | rplA_bact | ribosomal protein uL1 |
| TIGR00331 | hrcA | heat-inducible transcription repressor HrcA |
| TIGR02350 | prok_dnaK | chaperone protein DnaK |
| TIGR00096 | TIGR00096 | 16S rRNA (cytidine(1402)-2'-O)-methyltransferase |
| TIGR01044 | rplV_bact | ribosomal protein uL22 |
| TIGR00468 | pheS | phenylalanine--tRNA ligase, alpha subunit |
| TIGR00008 | infA | translation initiation factor IF-1 |
| TIGR03654 | L6_bact | ribosomal protein uL6 |
| TIGR00487 | IF-2 | translation initiation factor IF-2 |
| TIGR02348 | GroEL | chaperonin GroL |
| TIGR00485 | EF-Tu | translation elongation factor Tu |
| TIGR01164 | rplP_bact | ribosomal protein uL16 |
| TIGR00499 | lysS_bact | lysine--tRNA ligase |
| TIGR01067 | rplN_bact | ribosomal protein uL14 |
| TIGR00962 | atpA | ATP synthase F1, alpha subunit |
| TIGR00963 | secA | preprotein translocase, SecA subunit |
| TIGR01063 | gyrA | DNA gyrase, A subunit |
| TIGR01060 | eno | phosphopyruvate hydratase |
| TIGR00967 | 3a0501s007 | preprotein translocase, SecY subunit |
| TIGR01009 | rpsC_bact | ribosomal protein uS3 |
| TIGR01280 | xseB | exodeoxyribonuclease VII, small subunit |
| TIGR00651 | pta | phosphate acetyltransferase |
| TIGR00012 | L29 | ribosomal protein uL29 |
| TIGR00168 | infC | translation initiation factor IF-3 |
| TIGR02027 | rpoA | DNA-directed RNA polymerase, alpha subunit |
| TIGR00184 | purA | adenylosuccinate synthase |
| TIGR00521 | coaBC_dfp | phosphopantothenoylcysteine decarboxylase / ligase |
| TIGR00188 | rnpA | ribonuclease P protein component |
| TIGR00086 | smpB | SsrA-binding protein |
| TIGR00165 | S18 | ribosomal protein bS18 |
| TIGR00166 | S6 | ribosomal protein bS6 |
| TIGR00060 | L18_bact | ribosomal protein uL18 |
| TIGR00414 | serS | serine--tRNA ligase |
| TIGR00020 | prfB | peptide chain release factor 2 |
| TIGR00416 | sms | DNA repair protein RadA |
| TIGR02386 | rpoC_TIGR | DNA-directed RNA polymerase, beta' subunit |
| TIGR00459 | aspS_bact | aspartate--tRNA ligase |
| TIGR00755 | ksgA | ribosomal RNA small subunit methyltransferase A |
| TIGR03263 | guanyl_kin | guanylate kinase |
| TIGR00436 | era | GTP-binding protein Era |
| TIGR00928 | purB | adenylosuccinate lyase |
| TIGR00435 | cysS | cysteine--tRNA ligase |
| TIGR00418 | thrS | threonine--tRNA ligase |
| TIGR00653 | GlnA | glutamine synthetase, type I |
| TIGR01744 | XPRTase | xanthine phosphoribosyltransferase |
| TIGR03635 | uS17_bact | ribosomal protein uS17 |
| TIGR01534 | GAPDH-I | glyceraldehyde-3-phosphate dehydrogenase, type I |
| TIGR01059 | gyrB | DNA gyrase, B subunit |
| TIGR00337 | PyrG | CTP synthase |
| TIGR03594 | GTPase_EngA | ribosome-associated GTPase EngA |
| TIGR01021 | rpsE_bact | ribosomal protein uS5 |
| TIGR02075 | pyrH_bact | UMP kinase |
| TIGR00362 | DnaA | chromosomal replication initiator protein DnaA |
| TIGR00855 | L12 | ribosomal protein bL12 |
| TIGR00496 | frr | ribosome recycling factor |
| TIGR01050 | rpsS_bact | ribosomal protein uS19 |
| TIGR01171 | rplB_bact | ribosomal protein uL2 |
| TIGR01099 | galU | UTP--glucose-1-phosphate uridylyltransferase |
| TIGR02012 | tigrfam_recA | protein RecA |
| TIGR00580 | mfd | transcription-repair coupling factor |
| TIGR01049 | rpsJ_bact | ribosomal protein uS10 |
| TIGR01071 | rplO_bact | ribosomal protein uL15 |
| TIGR01029 | rpsG_bact | ribosomal protein uS7 |
| TIGR01073 | pcrA | ATP-dependent DNA helicase PcrA |
| TIGR00871 | zwf | glucose-6-phosphate dehydrogenase |
| TIGR00690 | rpoZ | DNA-directed RNA polymerase, omega subunit |
| TIGR00952 | S15_bact | ribosomal protein uS15 |
| TIGR01034 | metK | methionine adenosyltransferase |
| TIGR01011 | rpsB_bact | ribosomal protein uS2 |

Supplementary Table 3. GenBank accession numbers of strains used for phylogenetic comparison to *Weissella tructae* (Figure 4 and 5)

| **Strain** | **Accession** |
| --- | --- |
| *Fructobacillus pseudoficulneus* DSM 15468 | FNWS00000000.1 |
| *Weissella koreensis* KCTC 3621 | AKGG00000000.1 |
| *Weissella kandleri* DSM 20593 | NZ_JQBP00000000.1 |
| *Weissella cibaria* KACC 11862 | AEKT00000000.1 |
| *Weissella confusa* DSM 20196 | NZ_JQAY00000000.1 |
| *Weissella bombi* R-53094 | NZ_FMAO00000000.1 |
| *Weissella jogaejeotgali* FOL01 | [CP014332.1](https://www.ncbi.nlm.nih.gov/nuccore/CP014332.1/) |
| *Weissella paramesenteroides* ATCC 33313 | ACKU00000000.1 |
| *Weissella halotolerans* DSM 20190 | ATUU00000000.1 |
| *Weissella viridescens* NCDO 1655 | CYXF00000000.1 |
| *Weissella minor* DSM 20014 | NZ_JQCD00000000.1 |
| *Weissella ceti CECT 7719* | CP074441 |


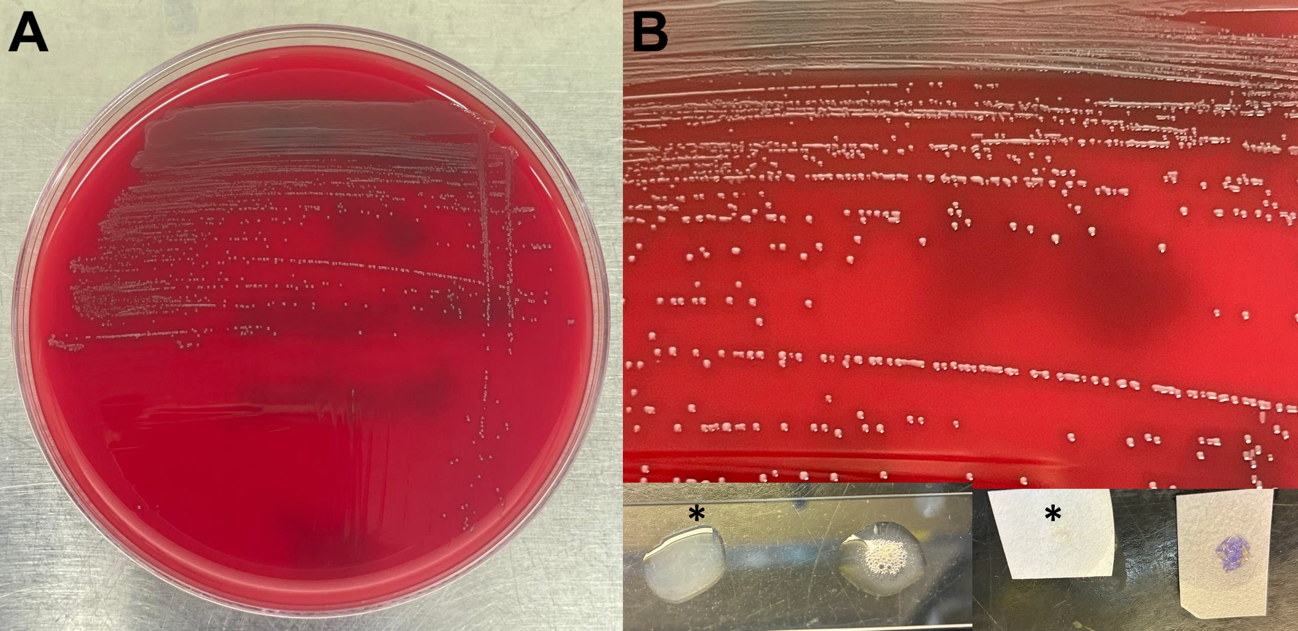


Supplementary Figure 1. Physiochemical characteristics of *Weissella tructae.* Colony morphology when grown overnight at 28 °C in trypticase soy agar with 5% sheep blood. The opaque, white colonies were 0.5 to 1.0 mm in diameter (A). Evaluated against a light source, distinct α-hemolytic activity was observed as a greenish halo surrounding colonies (B). *Weissella tructae* isolates were catalase and cytochrome oxidase-negative (asterisk), as evidenced by a lack of bubble formation and blue color change, respectively. *Aeromonas cavieae* was used for comparison (catalase and cytochrome oxidase-positive).
